# Quantitative CT Correlates with Local Inflammation in Lung of Patients with Subtypes of Chronic Lung Allograft Dysfunction

**DOI:** 10.3390/cells11040699

**Published:** 2022-02-16

**Authors:** Sundaresh Ram, Stijn E. Verleden, Alexander J. Bell, Benjamin A. Hoff, Wassim W. Labaki, Susan Murray, Bart M. Vanaudenaerde, Robin Vos, Geert M. Verleden, Ella A. Kazerooni, Stefanie Galbán, Charles R. Hatt, Meilan K. Han, Vibha N. Lama, Craig J. Galbán

**Affiliations:** 1 Department of Radiology, University of Michigan, Ann Arbor, MI 48109, USA; sundarer@med.umich.edu (S.R.); alejbell@med.umich.edu (A.J.B.); bahoff@med.umich.edu (B.A.H.); ellakaz@med.umich.edu (E.A.K.); sgalban@med.umich.edu (S.G.); 2 Antwerp Surgical Training, Anatomy and Research Centre (ASTARC), Faculty of Medicine and Health Sciences, University of Antwerp, Wilrijk, 2610 Antwerp, Belgium; stijn.verleden@uantwerpen.be; 3 Department of Chronic Diseases and Metabolism (CHROMETA), Katholieke Universiteit Leuven, 3000 Leuven, Belgium; bart.vanaudenaerde@kuleuven.be (B.M.V.); robin.vos@kuleuven.be (R.V.); geert.verleden@kuleuven.be (G.M.V.); 4 Division of Pulmonary and Critical Care Medicine, University of Michigan, Ann Arbor, MI 48109, USA; wlabaki@med.umich.edu (W.W.L.); mrking@med.umich.edu (M.K.H.); vlama@med.umich.edu (V.N.L.); 5 Department of Biostatistics, School of Public Health, University of Michigan, Ann Arbor, MI 48109, USA; skmurray@med.umich.edu; 6 Respiratory Division, University Hospital Leuven, 3000 Leuven, Belgium; 7 Imbio, LLC, Minneapolis, MN 55405, USA; charleshatt@imbio.com

**Keywords:** chronic lung allograft dysfunction, bronchiolitis obliterans syndrome, restrictive allograft syndrome, inflammation, computed tomography, parametric response mapping

## Abstract

Chronic rejection of lung allografts has two major subtypes, bronchiolitis obliterans syndrome (BOS) and restrictive allograft syndrome (RAS), which present radiologically either as air trapping with small airways disease or with persistent pleuroparenchymal opacities. Parametric response mapping (PRM), a computed tomography (CT) methodology, has been demonstrated as an objective readout of BOS and RAS and bears prognostic importance, but has yet to be correlated to biological measures. Using a topological technique, we evaluate the distribution and arrangement of PRM-derived classifications of pulmonary abnormalities from lung transplant recipients undergoing redo-transplantation for end-stage BOS (N = 6) or RAS (N = 6). Topological metrics were determined from each PRM classification and compared to structural and biological markers determined from microCT and histopathology of lung core samples. Whole-lung measurements of PRM-defined functional small airways disease (fSAD), which serves as a readout of BOS, were significantly elevated in BOS versus RAS patients (*p* = 0.01). At the core-level, PRM-defined parenchymal disease, a potential readout of RAS, was found to correlate to neutrophil and collagen I levels (*p* < 0.05). We demonstrate the relationship of structural and biological markers to the CT-based distribution and arrangement of PRM-derived readouts of BOS and RAS.

## 1. Introduction

Transplanted lungs have one of the highest rejection rates among all solid organ transplantations, and approximately 50% of lung transplant recipients suffer from chronic lung allograft dysfunction (CLAD) within five years of transplantation [1,2]. The main subtypes of CLAD are (a) bronchiolitis obliterans syndrome (BOS), which is characterized by persistent spirometric airflow obstruction and mostly air trapping on expiratory X-ray computed tomography (CT), with or without bronchiectasis, and (b) restrictive allograft syndrome (RAS), which is defined by decreased total lung capacity ≤90% of baseline and persistent radiographic opacities [3,4,5]. The underlying pathology of both subtypes of CLAD is irreversible fibroproliferation, either strictly airway-related in BOS, or involving both the airways and the parenchyma in RAS. In RAS, fibroproliferation leads to fibrotic remodeling of the lung, which is preceded by immune-mediated graft injury. Once persistent lung function loss occurs, prognosis is poor, with most patients demonstrating ongoing decline [6]. Of note, prognosis is significantly better for BOS than for RAS patients (3–5 years post-diagnosis versus 6–18 months).

We previously demonstrated using a combination of CT and microCT that obstruction of pre-terminal airways is common in both BOS and RAS (albeit significantly higher in more distal airways in BOS), while in RAS there is also parenchymal destruction leading to further distortion and remodeling of terminal bronchioles [7,8]. Lungs with end-stage RAS were characterized by a more pronounced inflammatory response with increased presence of neutrophils, eosinophils, mast cells, B cells and cytotoxic T cells compared to controls. In contrast, lungs with end-stage BOS only showed increased T and B cells, along with increased neutrophilia around the airways, compared to controls [9].

Parametric response mapping (PRM) is a quantitative CT-based algorithm that, when applied to paired inspiratory and expiratory high-resolution computed tomography (HRCT), has been shown to simultaneously detect parenchymal abnormalities associated with emphysema and small airways disease (SAD) in patients with chronic obstructive pulmonary disease (COPD) [10,11]. The ability of this technique to quantify SAD in COPD has been validated using microCT analysis of core samples from explanted lungs [12]. This study was the first to demonstrate the correlation of PRM-derived SAD to areas of lung tissue with terminal bronchial loss, luminal narrowing and obstruction as determined by microCT. In addition, PRM has been extended as a readout for other lung diseases, offering insights into pulmonary complications associated with chronic rejection from hematopoietic stem cell and lung transplantations [13,14,15,16]. This CT-based technique has recently been demonstrated to also offer valuable prognostic information for potential CLAD, identifying patients most at risk of developing CLAD or dying [17].

In these studies, PRM classifications, inherently three-dimensional (3D) datasets of pulmonary abnormalities, have been reported simply as percentages of lung volume. We have expanded the clinical application of PRM by demonstrating how topological analysis of PRM (tPRM) provides information on the distribution and arrangement of PRM classifications that correlates to clinically meaningful measures of COPD severity [18]. In this study, we extend tPRM to analysis of CLAD, as we investigate the relationship between topological features of PRM-derived classifications and structural and biological markers obtained from core samples extracted from explants of lung transplant recipients diagnosed with BOS and RAS who underwent re-transplantation or autopsy.

## 2. Materials and Methods

### 2.1. Patient Cohort

All patients who underwent double lung transplant reached a best postoperative forced expiratory volume at 1 s percent predicted (FEV1pp) > 80% and received azithromycin treatment for potential CLAD but were found to be nonresponsive. BOS and RAS were diagnosed according to the most recent International Society for Heart and Lung Transplantation guidelines. In brief, BOS patients showed a decline in FEV1pp > 20% compared to baseline but did not show evidence of concomitant restriction (no total lung capacity pp (TLCpp) < 90% or forced vital capacity pp (FVCpp) < 80% of baseline), without evidence of persistent opacities on CT. In contrast, RAS patients showed a simultaneous decrease in FEV1pp and TLCpp and persistent pleuroparenchymal opacities. Inspiratory and expiratory CT scans were acquired from all patients and the last CT scan prior to redo transplantation was used for further analysis. Clinical diagnosis was confirmed with pathological analysis of the contralateral lung with typical interstitial and (sub)pleural fibrosis in RAS and mostly inconspicuous parenchyma and airway obliteration in BOS. This study was approved by the local hospital’s ethical committee (S57752).

### 2.2. Computed Tomography

CT data were obtained as whole lung volumetric CT scans at full inspiration (TLC) and at relaxed expiration (functional residual capacity (FRC)) on Somatom scanner (Siemens, Munich, Germany) and reconstructed using a b60 or b70 reconstruction kernel. Slice thickness was 1 mm for all scans. All CT scans were checked for Hounsfield unit (HU) drift and if necessary corrected based on aortic blood (50 HU) and central air (1000 HU) as previously described [19].

### 2.3. Parametric Response Map

PRM was applied to all paired inspiratory and expiratory CT scans from both study groups. Briefly, lungs from paired scans were segmented from the thoracic cavity using an in-house algorithm written in MATLAB (The MathWorks, Inc., Natick, MA, USA). The whole-lung inspiration CT scan was spatially registered and aligned to the CT scan obtained at expiration using a deformable image registration algorithm provided in Elastix, an open-source image registration software package [20,21]. Once complete, the images share the same geometric space where each voxel, the smallest unit of volume in a 3D image dataset, consists of HU values at inspiration and expiration. To minimize the effects of noise on the PRM analysis, a 3 × 3 × 3 median filter was applied to both spatially aligned CT scans. Individual voxels were then classified based on predetermined thresholds as normal (color coded green), functional small airways disease (fSAD; color coded yellow), emphysema (color coded red), and parenchymal disease (color coded magenta). Voxels with values ≥ −950 HU and <−810 HU at inspiration and ≥−856 HU at expiration were classified normal, ≥−950 HU and <−810 HU at inspiration and <−856 HU at expiration were fSAD, <−950 HU at inspiration and <856 HU at expiration were emphysema, and ≥−810 HU at inspiration were parenchymal disease (PD) [13]. Global PRM measures were presented as relative lung volumes.

### 2.4. Topological Analysis of PRM (tPRM)

Topological properties of each PRM classification map were defined in this study through the Minkowski measures (local estimates of the Minkowski functionals) associated with 3D distributions: Volume (V, in mm^3^), Surface Area (S, in mm^2^), Mean Breadth (B, in mm), and the Euler–Poincaré characteristic (χ) [22]. A detailed description of these parameters is provided in the Supplementary Materials in [18]. Maps of Minkowski measures (i.e., V, S, B, and χ) were computed using a moving window of size 21^3^ voxels evaluated on a grid of every 5th voxel. V, S, and B were normalized by the Minkowski estimate of the mask within the same local window volume (rather than a direct calculation of the mask volume in the window as previously described) and χ by the masked window voxel count. Minkowski measures were quantified per subject as the mean local normalized value over the entire lung volume for group comparisons and regression. Final displayed representations of spatially resolved indices have been linearly interpolated back to original dimensions. It is important to note that the volume density of a given PRM classification is proportional to relative volumes of PRM classification. All image processing was performed using in-house algorithms developed in a technical computing language (MATLAB, The MathWorks Inc., Natick, MA, USA).

### 2.5. Sampling of the Ex Vivo Lung

Since 2009, explant lungs have been prospectively collected from all lung transplant patients undergoing lung redo transplantation or at autopsy (IRB approval UZ/KU Leuven S52174). Permission for CT analysis was requested separately from the UZ/KU Leuven ethics committee via approval number S57752. Explant lungs were cannulated and inflated to total lung capacity by applying a transpulmonary pressure of 30 cm H_2_O. They were then deflated to 10 cm H_2_O transpulmonary pressure, followed by freezing in the fumes of liquid nitrogen vapor. Lungs were scanned using HRCT (Siemens Definition Flash 120 kV; 110 mAs; Siemens, Munich, Germany). Following CT, the lungs were cut with a band saw, from apex to base, into slices of 2 cm and systematically sampled. Photographs were taken before and after sampling to ensure proper matching. Four cores per lung were selected for further analysis. These cores were chosen to present the different spectra of disease in the lungs. Specifically, for RAS patients, we included both fibrotic and non-fibrotic areas.

### 2.6. Topological PRM Analysis of Lung Cores

Topological features of individual cores were determined through spatial alignment of the in vivo paired CT scans to a photograph of the explanted cored lung section. This method was previously reported [12]. The process involved the following five steps. First, the pre-transplant inspiration CT scan was spatially aligned to the paired expiration CT scans using the Elastix deformable registration algorithm. Second, the lung used for the microCT analysis was segmented from the thoracic cavity in the pre-transplant expiration CT scan and spatially aligned to the CT scan of the explanted inflated frozen lung using the same deformable registration algorithm. The third step, registering the inflated lung to the uncored section RGB photograph, required additional post-processing. The photograph of the uncored section was converted to gray scale and subsampled by a factor of 10. A histogram equalization algorithm was applied to improve image contrast. To obtain the 3D topological maps for each PRM classification within the core, the image of the uncored lung section was replicated such that a 3D data set was constructed with the same number of slices as the inflated frozen lung 3D CT scan. The uncored lung section image on the center slice was then segmented. A similar transformation, i.e., rotate-translate-isotropic scaling, was performed to spatially align the inflated lung CT scan to the segmented uncored section in the dataset. Fourth, the uncored lung section was spatially aligned to the cored lung section using post-processing steps described in step three. Finally, the transformation matrices from each step were applied to the topological maps for each PRM classification so that each map (i.e., V, S, B and χ) was aligned to the cored section. Mean values for the topological features were calculated for the individual cores. The core volume of interest was determined by contouring the cored volume in the 3D cored lung section dataset. The number of contoured slices was determined by dividing the section thickness by the transformed voxel size (35 slices = 20 mm section (0.82 scaling factor × 0.7 mm slice thickness for inflated lung CT scan)).

### 2.7. MicroCT Imaging and Analysis of the Ex Vivo Lung Cores

The frozen cores were scanned in frozen condition with microCT (Skyscan 1172; Bruker, Kontich, Belgium) using a cooling stage (resolution 10 µm; 40 kV, 226 mA, 0.5° rotation steps). After scanning, the images were reconstructed with NRecon (Bruker, Kontich, Belgium) and processed using CT for assessment of the parenchymal morphometry (tissue percentage of core and surface density) and by manually counting the number of terminal bronchioles (TB/mL) [23]. A portion of the core was vacuum embedded in OCT (Sakura, Tokyo, Japan), and cryosections (8 µm) were made. Sections were stained for hematoxylin and picrosirius red (total collagen). Antibodies were used to detect collagen type I (ab6308, Abcam, Cambridge, UK) and collagen type III (2150-0100, Bio-Rad, Hercules, CA, USA). Detailed methods for histology labeling and processing steps are available in [24]. To assess the presence of relevant immune cells, immunohistochemical stains for T-lymphocytes (CD4, CD8), B-lymphocytes (CD20), neutrophils (MPO), eosinophils (ECP), mast cells (CD117), and macrophages (CD68) were made using an AEC chromogen. Slides were digitalized using an Aperio digital pathology slide scanner (Leica Biosystems Inc., Wetzlar, Germany) and analyzed using Qupath (version v0.3.2) and ImageJ (version 1.53o). Details on the methods used for immunohistochemical staining protocols are provided in Supplementary Table S1 and the analysis is described in [9]. The relative number of positively stained cells was calculated by normalizing stained cells to all identified cells.

### 2.8. Statistical Analysis

All data are presented as the mean ± the standard deviation unless stated otherwise. Differences in metrics between CLAD subtypes were assessed by Mann–Whitney U Test for all continuous variables and Fisher Exact test for categorical variables. Correlation analysis was determined using Spearman rho. All statistical computations were performed using a statistical software package (SPSS Software Products, version 28.0.1). Results were considered statistically significant at the two-sided 5% comparison-wise significance level (*p* < 0.05).

## 3. Results

### 3.1. Study Population

Patient characteristics are presented in Table 1. There were no significant differences in recipient age, gender, height, and weight. Subtle differences were observed in underlying disease between groups. FEV1pp (20.3 ± 4.0 and 30.2 ± 10.7 for BOS and RAS, respectively) and FEV1/FVC (0.42 ± 0.08 and 0.72 ± 0.20 for BOS and RAS, respectively) were found to be significantly lower in BOS than in RAS patients. Mean lung density as measured on CT at expiration was significantly lower in BOS patients (−806 ± 55 HU) compared with RAS patients (−678 ± 112 HU).

### 3.2. Representative Cases

**Figure 1:**
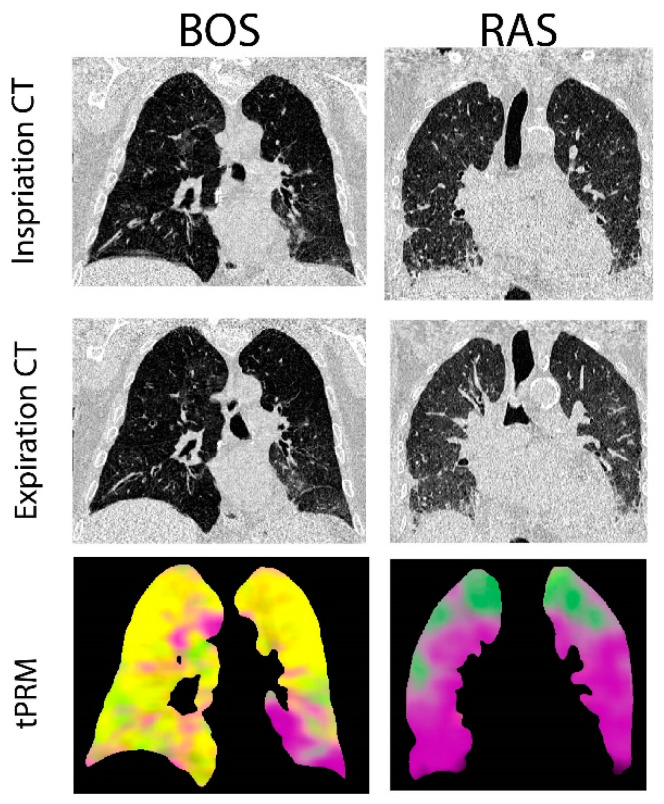
Representative cases diagnosed with BOS and RAS. Presented are the coronal view of CT scans acquired at inspiration and expiration with corresponding topological parametric response mapping (tPRM)-derived volume density maps for normal parenchyma (green), functional small airways disease (fSAD; yellow) and parenchymal disease (PD; magenta).

Provided in Figure 1 are CT coronal views of the thoracic region with lungs at full inspiration and expiration for a patient with BOS and one with RAS. The BOS case is a male diagnosed with BOS 206 days post-transplant. This patient had a FEV1pp of 17% and FEV1/FVC of 0.4 when undergoing second transplantation. The RAS case is a female diagnosed with RAS 2649 days post-transplant. This patient had a FEV1pp of 50% and FEV1/FVC of 0.88 when undergoing second transplantation. The corresponding volume density maps for PRM-derived normal parenchyma (green), fSAD (yellow) and PD (magenta) are also presented. The lung transplant recipient who developed BOS had more PRM-derived fSAD distributed throughout the lungs, whereas the patient who developed RAS had more PRM-derived PD (Figure 1).

### 3.3. Group Comparison of tPRM Whole Lung Analysis

**Figure 2:**
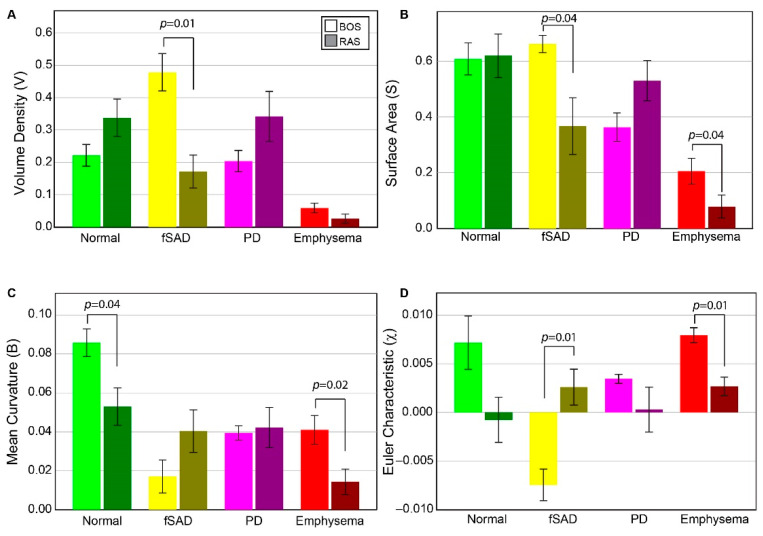
Chronic lung allograft dysfunction (CLAD) subtype group comparison of all tPRM metrics. Barplots are presented as the mean and standard error of the mean for topological measures across both BOS (N = 6) and RAS (N = 6), for PRM^Norm^ (green), PRM^fSAD^ (yellow), PRM^Emph^ (red) and PRM^PD^ (magenta). BOS = Bronchiolitis Obliterans Syndrome, RAS = Restrictive Allograft Syndrome, Norm = normal parenchyma, fSAD = functional small airways disease, Emph = emphysema, PD = parenchymal disease. (**A**) Volume density, describing class magnitude (relative amounts of voxels). (**B**) Surface area, describing class exposure (exposed faces of voxels). (**C**) mean breadth, which can be interpreted as average curvature (convex/concave) of class surfaces. (**D**) Euler–Poincaré characteristic, describing class homology, determined by number and type of holes within class volumes. The legend indicates measures from BOS and RAS as light and dark shades, respectively, for a given PRM color-code.

Volume density, surface area, and Euler–Poincaré characteristic metrics associated with PRM-derived fSAD were found to differ significantly between BOS and RAS (Figure 2). The volume density of fSAD, a readout of disease extent (V^fSAD^), was on average more than double in BOS patients (0.48 ± 0.14) than in RAS patients (0.17 ± 0.13). The surface area, a readout of disease distribution (S^fSAD^), was also found to be elevated in the BOS group (0.66 ± 0.08 mm^−1^) compared to RAS (0.37 ± 0.25 mm^−1^), suggesting most patients have a diffuse spread of BOS throughout the lungs. Euler–Poincaré characteristic (χ^fSAD^), a readout of disease pockets or holes, was negative for the BOS group (−0.007 ± 0.004). Negative values in χ^fSAD^ occur when PRM-derived fSAD is the dominant classification within the lungs with holes that may comprise of other PRM classes, or structures such as airways and blood vessels. In contrast, the observed mean value of χ^fSAD^ for RAS patients was positive (0.003 ± 0.001), which is associated with small pockets of PRM-derived fSAD. Although the mean curvature of PRM-derived fSAD, a readout of tunneling within this classification, was higher in RAS (0.04 ± 0.01 mm^−2^) than in BOS patients (0.02 ± 0.02 mm^−2^), this was not found to be significant.

In contrast to PRM-derived fSAD, the topology of PD did not result in significant differences between groups for any of the metrics. Notwithstanding, the volume density and surface area of PRM-derived PD generated expected trends. For V^PD^ and S^PD^, patients with RAS (0.34 ± 0.19 and 0.53 ± 0.18 mm^−1^, respectively) had higher values compared to patients with BOS (0.20 ± 0.03 (*p* = 0.145) and 0.36 ± 0.12 mm^−1^ (*p* = 0.091), respectively). Near identical values in mean curvature were observed between groups (BOS at 0.04 ± 0.01 mm^−2^ and RAS at 0.04 ± 0.03 mm^−2^). Although the mean value of χ^PD^ was lower in RAS (0.0003 ± 0.006) than in BOS (0.003 ± 0.001) patients, no significant difference (*p* = 0.132) was observed due to the large spread in values for RAS patients.

PRM-derived normal and emphysema provided mixed results. Only mean curvature for PRM-derived normal parenchyma showed a significant difference between groups (Figure 2). PRM-derived emphysema was found to generate significant differences between BOS and RAS for surface area, mean curvature and Euler–Poincaré characteristic. The volume density of PRM-derived emphysema on average was around 0.05 or less for all cases (0.05 is equivalent to 5% emphysema of total lung volume). This is partly attributed to scanner noise and airways that were not adequately segmented from the lung volume.

### 3.4. Correlation of Whole-Lung tPRM Metrics and PFT

Based on whole-lung analysis of individual tPRM metrics, the volume density of PRM-derived fSAD and PD were elevated for BOS and RAS, respectively. Using the total study population (N = 12), correlations to pulmonary function measurements were assessed for all tPRM metrics of these two PRM classifications (Table 2). FVC and FEV1/FVC were strongly correlated to multiple tPRM metrics, except mean curvature (B) for fSAD. As PRM-derived fSAD serves as a readout of BOS, patients with BOS typically manifest higher levels of FVC and fSAD than their RAS counterparts. The strongest correlation was observed for FVC against χ^fSAD^ (R = −0.848, *p*-value < 0.001). Here, patients with BOS had higher FVC and negative χ^fSAD^, whereas RAS patients, except for one, had low FVC and positive χ^fSAD^. V^fSAD^ and S^fSAD^ demonstrated significant correlations for FVC and FEV1/FVC (Table 2). For PRM-derived PD, only the surface area (S) was found to result in significant correlates (FVC: R = −0.734, *p*-value = 0.006 and FEV1/FVC: R = 0.615, *p*-value = 0.033). Nevertheless, V^PD^ and χ^PD^ demonstrated mild correlations to FVC with *p*-values equal to 0.07 and 0.08, respectively. This trend was not observed when correlated to FEV1/FVC (*p*-values = 0.175 for V^PD^ and 0.152 for χ^PD^).

### 3.5. tPRM Metrics from Cored Samples

**Figure 3:**
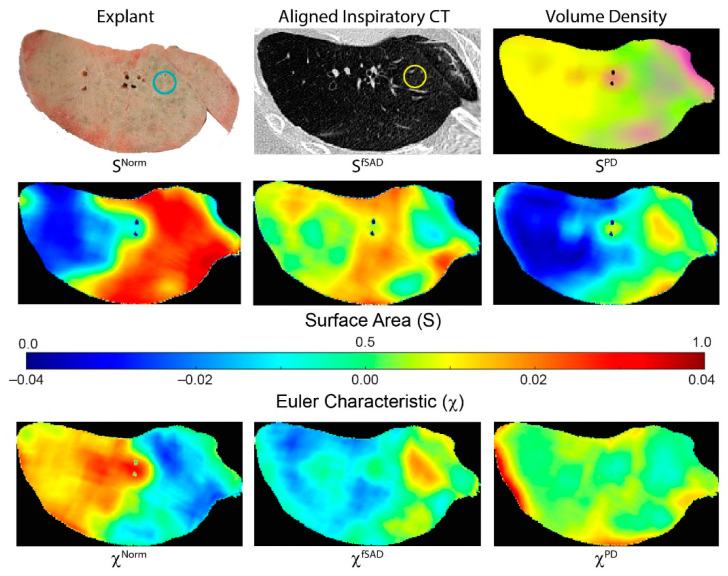
Representative case illustrating spatial heterogeneity in tPRM measures. Presented is a photo of an explanted lung section, with corresponding aligned inspiratory CT scan and tPRM-derived volume density map, surface area and Euler–Poincaré characteristic. Volume density map depicts the local extent of normal parenchyma (green), fSAD (yellow) and PD (magenta). Surface area and Euler–Poincaré characteristic are provided for each PRM classification. The circles on explant (blue) and CT (yellow) provide approximate location and size of an example core sample. This case is a male diagnosed with BOS 447 days post-transplant. This patient had a FEV1pp of 18% and FEV1/FVC of 0.41 when undergoing redo transplantation.

Figure 3 illustrates how tPRM metrics for individual cores depend on the location of the lung sample (core location provided on explant (blue circle) and CT scan (yellow circle)). This case is a male diagnosed with BOS 447 days post-transplant. This patient had a FEV1pp of 18% and FEV1/FVC of 0.41 when undergoing second transplantation. The distribution and arrangement of each PRM-derived classification generates topological measures that are spatially dependent on the underlying condition of the lung parenchyma. This lung section, approximately midway along the vertical axis (head to foot), comprised primarily of fSAD (yellow on volume density map) and normal parenchyma (green on volume density map). Regions with extreme values in volume density (low and high) resulted in low values of surface area (blue on surface area maps). Lung regions with a diffuse distribution of PRM classes resulted in higher values in S (red on surface area maps). This is clear for S^Norm^ where normal lung parenchyma regionally coexists with local parenchyma classified as fSAD and PD (Figure 3 S^Norm^). The low values in S^Norm^ were a consequence of that entire lung region being classified as fSAD. The Euler–Poincaré characteristic provides an indication of the dominance of any PRM class, as well as its transition to or from another PRM class. As seen for PRM-derived fSAD, χ^fSAD^ generated negative values where V^fSAD^ was highest. Within that same lung region, χ^Norm^ had positive values, suggesting an inferior contribution to this space (red regions). These trends are consistent with the whole lung analysis for χ^Norm^ and χ^fSAD^ in the BOS group (Figure 2).

### 3.6. tPRM Metrics of Individual Cores

**Figure 4:**
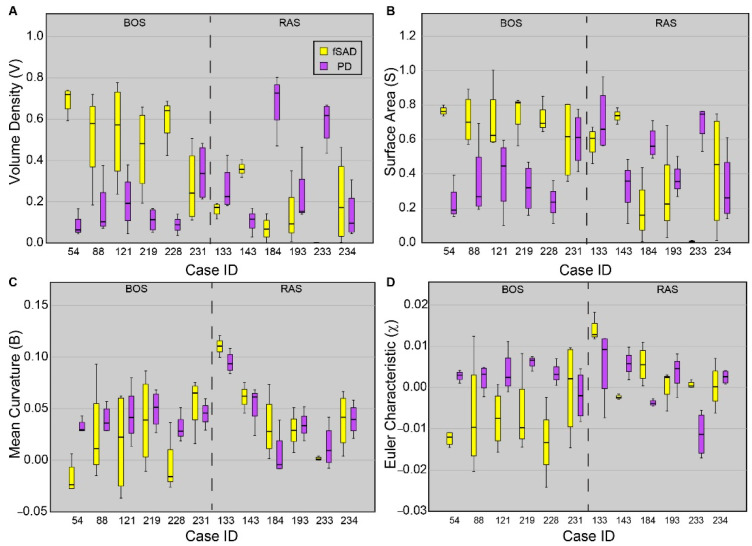
Boxplots for all four tPRM metrics of the dominant readouts from each patient. Presented is the (**A**) volume density, (**B**) surface area, (**C**) mean breadth, and (**D**) Euler–Poincaré characteristic, for fSAD (yellow) and PD (purple). Approximately four cores were acquired from each patient. Box lines represent the lower quartile, median, and upper quartile, and whiskers represent minimum and maximum values. Cases were separated based on disease subtype.

Topological values averaged over each core are presented for all cores sampled from each patient (Figure 4). As PRM-derived fSAD and PD are readouts of BOS and RAS, respectively, only these PRM classes are provided. Five out of the six patients diagnosed with BOS were found to have cores with V^fSAD^ > V^PD^, with median values of V^fSAD^ around or greater than 0.5 (i.e., 50% of the core volume was identified as fSAD). Only one case (231) was found to have similar contributions of PRM-derived fSAD (median V^fSAD^ = 0.24) and PD (median V^PD^ = 0.34) within the core sample. The surface area (S) and Euler-Poincaré characteristic (χ) were also consistent among BOS cases, except for 231. Unlike the BOS patients, no consistent topology of fSAD and PD were identified from the core samples obtained from lung sections of RAS patients. Two of the six RAS patients had core samples with elevated levels of V^PD^ with median values above 0.6. Three of the six (cases 133, 193, and 234) had similar volume densities for fSAD and PD. The sixth case (143) had higher values of V^fSAD^ than V^PD^, like trends observed in BOS patients.

### 3.7. MicroCT and Histopathology Analysis

**Figure 5:**
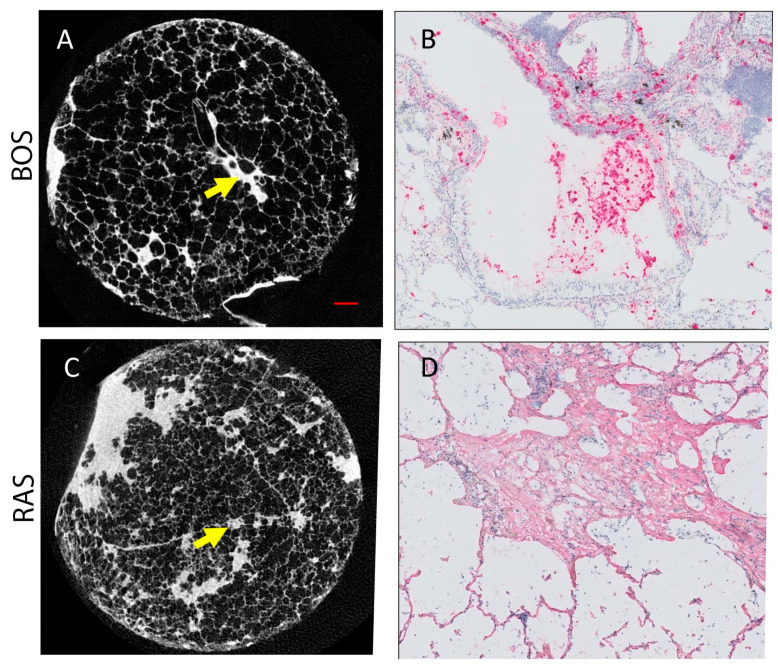
Representative microCT and immunohistochemistry for BOS ((**A**,**B**), respectively) and RAS ((**C**,**D**), respectively) cases. (**A**) MicroCT image of BOS core where normal airway parenchyma is seen. The arrow indicates an obliterated airway. (**B**) Neutrophil staining (pink) corresponds to the site of the arrow on (**A**). (**C**) MicroCT image of RAS core where some patchy fibroblast foci can be seen. (**D**) Collagen I stain (pink) is shown at the site of the arrow on (**C**). Scale bar (red line in (**A**)) represents 1 mm on both microCT (**A**,**C**). (**B**,**C**) were counter stained with hematoxylin (blue).

Figure 5 presents microCT and immunohistochemistry stains of representative cores from patients diagnosed with BOS (Figure 5A,B) and RAS (Figure 5C,D). Morphologically inconspicuous lung parenchyma can be seen on microCT with segmental airway scarring and obstruction (Figure 5A). Representative slides for all stains for each case and a control are provided in Supplementary Figure S1. In contrast, the microCT of the RAS patient demonstrated loss of alveolar space, opacities, and extensive scarring of the interstitium (Figure 5C). Infiltration of MPO-expressing cells was evident in the airways for the BOS patient (Figure 5B). Collagen I (COL I) deposits were identified in the interstitial space of the RAS patient (Figure 5D). Group comparison of the microCT measures found significant differences in the tissue percentage of the cores and surface density (Table 3). The BOS group was found to have significantly lower levels of CD4+ T cells than the RAS group (Table 3). Although not significant (*p* = 0.06), neutrophil levels were more elevated in BOS than RAS subjects.

### 3.8. Correlation of tPRM Metrics to Structural and Biological Markers

For PRM classes fSAD and PD, correlations were determined for topology metrics and structural and biological markers (Table 4). This analysis was repeated using pooled core data (N = 45) and core data from each group (N = 23 for BOS and N = 22 for RAS). Combining all cores from each group resulted in significant correlations (yellow text in Table 4 indicates *p*-value < 0.05) of tissue percentage of cores (T%) and surface density (SD) to nearly all the topology measures for PRM-derived fSAD. The strongest correlation was observed between V^fSAD^ and T% (R = −0.599, *p* < 0.001). PRM-derived PD was also found to demonstrate significant correlations with the volume density (V^PD^) to T% (R = 0.457, *p* = 0.002) and the number of terminal bronchioles per mL (TB/mL) (R = 0.380, *p* = 0.010). MPO levels were found to correlate significantly to the volume density and surface area (S) of both PRM classes, as well as to the Euler–Poincaré characteristic of PD (χ^PD^). CD117 was found to correlate with V and S of fSAD and COL I with χ^PD^.

Correlations for individual groups generated different results from the pooled data. No significant correlations were observed within the BOS group. Nevertheless, non-significant correlations for BOS subjects were seen for V^PD^ and TB/mL (R = 0.363, *p* = 0.089). Cores obtained from RAS patients showed significant correlations between tPRM metrics and structural and biological markers. Like the pooled data, TB/mL, MPOs, and COL I were found to significantly correlate with various topology metrics from both PRM classes. The strongest correlation, irrespective of pooling, was observed between V^PD^ and MPO levels, with a R of −0.684 and *p*-value < 0.001.

## 4. Discussion

In this study, we confirmed the role of PRM-derived fSAD and PD as CT-based readouts of BOS and RAS, respectively, in lung transplant recipients. We also demonstrated the relationship between the extent, distribution, and arrangement of our PRM-derived classifications of pulmonary abnormalities, and the structural and biological microenvironment of lung parenchyma in these same CLAD subtypes. We provide further support for our findings by demonstrating elevated levels of fSAD and PD in BOS and RAS patients, respectively, and a correlation between topological measures of fSAD and PD and microCT-measured surface density and MPO levels in cored lung samples.

An important find in our study was confirmation that PRM-derived classifications are strongly associated with clinically relevant measures of CLAD subtypes. Consistent with prior publications by both our groups [13,14,17,25] and researchers from Stanford University [15], the extent of PRM-derived fSAD, as measured by its volume density, was observed to be a readout of BOS. In our study, V^fSAD^ was found to be on average around 0.48 ± 0.18 of the total lung volume. This is equivalent to saying 48% of the lungs are PRM-derived fSAD. This value was higher than the ~30% reported previously, which has been demonstrated as a link to prognosis [14,15,16]. As described in the Methods section, the mean of V^fSAD^ over the lungs is equal to the percentage of PRM-derived fSAD normalized to 100. Taking this into account, we attribute the differences in the extent of fSAD reported here and in earlier studies to the severity of the disease and not to differences in calculation. Unlike the previous studies where PRM was determined at the time of diagnosis, patients in our cohort were scheduled for second lung transplants due to end-stage CLAD. With respect to the extent of PRM-derived PD, elevated values found in RAS patients (Figure 2) were consistent with previously published work [14,15,17]. Despite this consistency, wide variability in measurements occurs across the literature, likely due to differences in the time of measurement as part of the study designs, as well as to the inherent variability in the degree and location of fibrosis which has been previously discussed [26].

Case-specific and spatial variability in topological features within cores were evident in our study population. Between the cohorts, the lung transplant recipients diagnosed with BOS had similar topological values of volume density (V), surface area (S) and Euler–Poincaré characteristic (χ) for PRM-derived fSAD and PD. Out of the 6 BOS cases, 5 were found to have large differences in the median values over all cores per case in fSAD and PD. The cores from these cases had extensive small airways disease (V^fSAD^ > V^PD^) that was highly diffuse (elevated S^fSAD^) and was the dominant pathology within the cores (i.e., fSAD) (negative χ^fSAD^) (Figure 4). This is consistent with our observation over whole lungs (Figure 2), where significant differences were only observed between BOS and RAS for V, S and χ. A large range of topology values between case-specific cores was observed, as depicted by the whiskers in Figure 4. These findings suggest local variability in the distribution and arrangement of disease subtypes within individual cases.

A unique trend in topological features could not be identified in lung transplant recipients diagnosed with RAS. Only cases 184 and 233, as seen in Figure 4, generated elevated V^PD^ and S^PD^ and negative χ^PD^, which is like the topology of PRM-derived fSAD in BOS patients. Further, case 143, who had radiographically-identified scarring, presented several similarities consistent with the BOS group. Among these were trends in V, S and χ of the core samples, and volume densities of 0.37 for fSAD and 0.11 for PD when averaged over the lungs. This patient also had FVC (L) and FEV1/FVC of 1.95 L and 0.68, respectively, again consistent with BOS. It is important to note that PRM is defined within a range of −1000 HU to −250 HU on both the inspiration and expiration CT scans. Unlike air trapping due to BOS, pulmonary fibrosis, a hallmark of RAS, may have densities well outside this range. As such, PRM only detects and quantifies parenchyma with mild opacities that may be attributed to pneumonitis [15] and fibrosis [14], which is the likely explanation for the observed discrepancy.

Topology measures for PRM-derived fSAD and PD demonstrated strong correlations to microCT and biological measures when CLAD subtypes were pooled (N = 45). The most pronounced correlations for microCT and biology to topologies were identified in percentage of core tissue and MPOs (Table 4). These findings changed when analyzing cores from specific CLAD subtypes. All significant correlations observed in the pooled analysis were lost in the BOS cohort. We attribute the lack of correlation in our topology measures to microCT and biological measures to the limited range in disease severity between the BOS cases. As shown in Figure 4 and discussed above, 5 out of 6 cases had very similar values in topological features for both PRM-derived disease classifications (i.e., fSAD and PD). In contrast, the RAS group presented significant correlations. Most notable was surface area of PRM-derived fSAD, which correlated with terminal bronchiole count/mL and MPO levels, and volume density of PRM-derived PD, which was found to correlate with MPO and COL I levels (Table 4). The retention of these significant finds from the pooled analysis is attributed to wide variability in disease subtypes found in the RAS cohort, which provided more dynamic range in CT, microCT and biological measures. We suspect that infiltration of MPO-expressing cells occurs prior to collagen deposits associated with interstitial scarring. This would explain not only the negative correlation between V^PD^ and MPOs (R = −0.684, *p* < 0.001) but also the positive correlation between V^PD^ and COL I levels (R = 0.478, *p* = 0.028).

Our study was subject to some limitations worth addressing. First, the number of cases (N = 12) accrued as part of this study limited our statistical power. Nevertheless, whole-lung analysis of our cohort generated volume densities of PRM-derived fSAD and PD (Figure 2) consistent with PRM results from earlier studies, specifically elevated fSAD and PD for BOS and RAS, respectively [14,15,17]. Another limitation of the small number of cases was that core measurements, around 3–4 cores per case, were treated as independent measures. Though variations between cases were observed, especially within the RAS group, large intra-case variability occurred as identified by large whiskers in Figure 4. We also acknowledge that we could not mark 100% of all cells for proper quantification as could be done by FACS analysis. However, the current study does resemble and very closely approaches the global picture of the presence and amount of the different immune cells and collagen. Finally, we recognize the potential errors associated with spatially aligning clinical volumetric CT scans to images of explanted lung sections. The process requires three transformations, each producing some degree of misalignment. Nevertheless, we have previously demonstrated this approach validates PRM-derived fSAD as a measure of bronchiolitis in COPD patients [12]. Like all experimental techniques, great care was taken to minimize variability in final measurements while providing a consistent and reproducible technique to correlate clinical CT scans to microCT and biological core measurements.

## 5. Conclusions

We have offered a view of the PRM landscape in a group of lung transplant recipients who developed specific CLAD subtypes. We identified a connection between the structural and biological makeup of the lung microenvironment and the topology of a clinically relevant CT-based readout of BOS and RAS. Our investigative group is working on developing additional techniques to elucidate the nature of small airway and parenchymal abnormality identified by PRM topology. These steps could further confirm the potential use and benefit of PRM for monitoring CLAD progression in lung transplant recipients.

## Figures and Tables

**Table 1:** Subject characteristics.

Measurements	BOS	RAS	*p*-Value
Number of Patients, n	6	6	
Donor age, years	34 (11)	45 (13)	0.09
Recipient age at explant, years	47 (14)	49 (16)	
Recipient gender, n (%)			
Male	2 (33%)	3 (50%)	
Female	4 (67%)	3 (50%)	
Recipient height, cm	170 (5)	172 (13)	
Recipient weight, kg	59 (13)	59 (11)	
Underlying disease, n (%)			
A1ATD	0 (0%)	1 (17%)	
CF	3 (49%)	1 (17%)	
COPD	1 (17%)	2 (32%)	
CHP	1 (17%)	0 (0%)	
IPF	1 (17%)	0 (0%)	
PHT	0 (0%)	1 (17%)	
Systemic Sclerosis + PHT	0 (0%)	1 (17%)	
FEV1 at CT, L	0.64 (0.11)	0.87 (0.36)	
FEV1pp at CT, (%)	20.3 (4.0)	30.2 (10.7)	**0.04**
FVC at CT, L	1.6 (0.6)	1.2 (0.4)	
FVCpp at CT, (%)	41.7 (11.7)	31.3 (10.0)	
FEV1/FVC at CT	0.42 (0.08)	0.72 (0.20)	**0.01**
CT lung volume, L			
Expiration	3.7 (1.3)	2.3 (0.9)	0.07
Inspiration	4.1 (1.4)	3.1 (1.1)	
CT mean lung density, HU			
Expiration	−806 (55)	−678 (112)	**0.03**
Inspiration	−826 (34)	−754 (73)	0.09

Continuous variables presented as mean (standard deviation). Fisher’s Exact test was used to compare sex. Mann–Whitney U Test was used for continuous data. *p*-values less than 0.1 are provided with significant values bolded (*p* < 0.05). BOS, bronchiolitis obliterans syndrome; RAS, restrictive allograft syndrome; A1ATD, alpha-1-antitrypsin deficiency; CF, cystic fibrosis; COPD, chronic obstructive pulmonary disease; CHP, chronic hypersensitivity pneumonitis; IPF, idiopathic pulmonary fibrosis; PHT, pulmonary hypertension; FEV1, forced expiratory volume at 1 s; FEV1pp, FEV1 percent predicted; FVC, forced vital capacity; CT, computed tomography; HU, Hounsfield Unit.

**Table 2:** Correlations of whole lung tPRM metrics of dominant phenotypes to pulmonary function measurements.

Metric	FEV1	FEV1pp	FVC	FVCpp	FEV1/FVC
V^fSAD^	−0.196	−0.504	0.666	0.301	−0.825
S^fSAD^	−0.007	−0.490	0.680	0.217	−0.615
B^fSAD^	0.000	0.198	−0.392	−0.462	0.413
χ^fSAD^	−0.112	0.374	−0.848	−0.594	0.727
V^PD^	−0.084	0.413	−0.543	−0.231	0.420
S^PD^	−0.140	0.307	−0.743	−0.434	0.615
B^PD^	−0.098	−0.367	−0.168	−0.385	0.196
χ^PD^	0.007	−0.490	0.522	0.098	−0.441

Notes: Presented are the correlation coefficients (R) obtained using Spearman rho on pooled data (N = 6 BOS and 6 RAS). Conditional formatting from red (min value) to blue (max value) through white (median value) has been applied over the entire table. Statistical significance was assessed at *p* < 0.05 and is indicated with yellow font.

**Table 3:** Structural and Biological Measures from Core Samples.

	Measures	BOS	RAS	*p*-Value
	Number of Cores, n	23	22	
	Mean number of cores per patient, n	3.8	3.7	
	Core Volume, mL	1.9 × 10^0^ (2.9 × 10^−1^)	2.0 × 10^0^ (4.0 × 10^−1^)	
**Structural**	Tissue Percentage of Core, %	2.9 × 10^1^ (5.3 × 10^0^)	5.2 × 10^1^ (1.9 × 10^1^)	**<0.001**
	Surface density, µm^−1^	1.3 × 10^−2^ (1.8 × 10^−3^)	8.9 × 10^−3^ (3.1 × 10^−3^)	**<0.001**
	Number of terminal bronchioles per volume, (n/mL)	4.2 × 10^0^ (1.9 × 10^0^)	4.9 × 10^0^ (2.2 × 10^0^)	
**Biological**	B cells (CD20)	3.1 × 10^−3^ (8.1 × 10^−3^)	3.2 × 10^−3^ (9.0 × 10^−3^)	
	Neutrophils (MPO)	4.3 × 10^−2^ (3.1 × 10^−2^)	3.0 × 10^−2^ (3.1 × 10^−2^)	0.06
	Mast cells (CD117)	6.1 × 10^−5^ (1.1 × 10^−4^)	1.3 × 10^−4^ (2.0 × 10^−4^)	
	Macrophages (CD68)	3.4 × 10^−2^ (2.7 × 10^−2^)	3.9 × 10^−2^ (2.9 × 10^−2^)	
	CD4+ T cells	5.5 × 10^−4^ (1.7 × 10^−3^)	1.3 × 10^−3^ (2.1 × 10^−3^)	**0.02**
	CD8+ T cells	4.1 × 10^−3^ (7.3 × 10^−3^)	3.5 × 10^−3^ (4.6 × 10^−3^)	
	Eosinophils (ECP)	6.3 × 10^−3^ (1.2 × 10^−2^)	4.3 × 10^−3^ (7.7 × 10^−3^)	
	Collagen I	1.6 × 10^−3^ (1.3 × 10^−3^)	2.2 × 10^−3^ (2.1 × 10^−3^)	
	Collagen III	1.2 × 10^−2^ (1.8 × 10^−2^)	1.9 × 10^−2^ (4.1 × 10^−2^)	

Continuous variables presented as mean (standard deviation). Group comparisons were performed using Mann–Whitney U test. Statistical significance was assessed at *p* < 0.05. *p*-values less than 0.1 are provided with significant values bolded. Tissue percentage of Core (%) was determined by normalizing the tissue volume to the core volume times 100. Biological measurements were determined by normalizing the number of positively stained cells to all identified cells.

**Table 4:** Correlations of tPRM metrics of dominant subtypes to structural and biological measurements.

	Metric	T%	SD	TB/mL	CD20	MPO	CD117	CD68	CD4	CD8	ECP	COL I	COL III
Pooled (N = 45)	V^fSAD^	−0.599	0.448	−0.282	−0.115	0.342	−0.310	−0.037	−0.171	−0.225	−0.036	−0.198	0.207
	S^fSAD^	−0.445	0.309	−0.270	−0.062	0.450	−0.313	0.059	−0.060	−0.135	0.031	−0.287	0.167
	B^fSAD^	0.262	−0.315	−0.090	0.121	0.123	0.013	0.037	0.181	0.148	−0.004	−0.104	−0.101
	χ^fSAD^	0.479	−0.453	0.099	0.118	−0.265	0.184	−0.049	0.171	0.119	−0.048	0.146	−0.161
	V^PD^	0.457	−0.236	0.380	−0.062	−0.470	0.262	0.074	0.069	0.088	0.050	0.292	−0.076
	S^PD^	0.425	−0.249	0.287	−0.020	−0.330	0.233	0.022	0.088	0.217	0.027	0.151	−0.081
	B^PD^	−0.063	−0.040	−0.095	0.088	0.274	−0.135	−0.104	0.159	0.190	−0.093	−0.260	−0.018
	χ^PD^	−0.209	−0.023	−0.231	0.111	0.374	−0.241	0.060	0.154	0.035	−0.043	−0.352	0.020
BOS (N = 23)	V^fSAD^	−0.304	−0.070	−0.132	−0.045	−0.106	−0.181	0.119	0.122	−0.225	−0.008	−0.070	0.307
	S^fSAD^	0.030	−0.121	−0.002	−0.084	0.289	−0.206	0.242	0.157	−0.054	0.063	−0.261	0.157
	B^fSAD^	0.292	−0.006	0.125	0.011	0.125	0.075	−0.145	−0.119	0.106	−0.100	0.109	−0.299
	χ^fSAD^	0.036	−0.015	0.053	−0.052	−0.060	0.091	−0.242	−0.248	−0.103	−0.207	0.278	−0.310
	V^PD^	0.148	0.283	0.363	−0.013	0.018	0.150	−0.112	−0.103	0.112	0.013	0.124	−0.236
	S^PD^	0.227	0.209	0.320	0.031	0.040	0.108	−0.093	−0.054	0.137	−0.001	0.126	−0.252
	B^PD^	0.130	0.050	0.169	−0.053	0.104	−0.018	−0.113	−0.053	0.147	−0.106	0.063	−0.268
	χ^PD^	−0.045	−0.128	−0.125	−0.115	0.116	0.010	0.098	0.017	0.221	−0.092	−0.296	−0.039
RAS (N = 22)	V^fSAD^	−0.119	−0.078	−0.398	0.182	0.399	−0.312	0.020	0.169	−0.149	0.119	−0.275	0.135
	S^fSAD^	−0.129	−0.115	−0.460	0.162	0.460	−0.297	0.054	0.180	−0.085	0.098	−0.327	0.133
	B^fSAD^	0.053	−0.394	−0.459	0.115	0.319	−0.247	0.106	0.314	0.083	−0.019	−0.380	0.212
	χ^fSAD^	0.224	−0.403	0.029	−0.004	−0.141	−0.054	−0.018	0.177	−0.020	−0.189	−0.067	0.072
	V^PD^	0.217	−0.156	0.328	−0.225	−0.684	0.270	0.130	−0.112	−0.057	−0.012	0.478	0.124
	S^PD^	0.188	−0.266	0.136	−0.117	−0.346	0.252	0.022	0.036	0.337	−0.111	0.092	0.097
	B^PD^	−0.097	−0.224	−0.273	0.259	0.420	−0.206	−0.058	0.404	0.283	−0.063	−0.491	0.119
	χ^PD^	−0.238	−0.049	−0.247	0.311	0.390	−0.419	0.031	0.305	−0.158	0.060	−0.289	0.106

Presented are the correlation coefficients (R) obtained using Spearman rho on pooled data from cores (N = 23 BOS and 22 RAS). T%, Tissue percentage of core; SD, Surface density; TB/mL, Number of terminal bronchioles per volume; CD20, B cells; MPO, Neutrophils; CD117, Mast cells; CD68, Macrophages; CD4, CD4+ T cells; CD8, CD8+ T cells; ECP, Eosinophils; COL I, Collagen I; COL III, Collagen III. Conditional formatting from red (min value) to blue (max value) through white (median value) has been applied over the entire table. Statistical significance was assessed at *p* < 0.05 and is indicated with yellow font.

## Data Availability

The data presented in this study are available on request from the corresponding author. The data are not publicly available in order to protect the privacy of the subjects of this study.

## Supplementary Materials

The following are available online at https://www.mdpi.com/article/10.3390/cells11040699/s1, Figure S1: Representative Slides for Each Stain, Table S1: Antibodies and Staining Protocols.
