# Supplementary material for: Quantitative CT Correlates with Local Inflammation in Lung of Patients with Subtypes of Chronic Lung Allograft Dysfunction
Source: Cells. 2022 Feb 16;11(4):699. doi: 10.3390/cells11040699 (PMC8870691; doi:10.3390/cells11040699)
Supplement: Supplementary file 1 [file cells-11-00699-s001.zip › cells-1513770-supplementary.pdf]

Supplementary Materials

**Item**	**Title/Description**
Supplementary Table S1	Antibodies and Staining Protocols
Supplementary Figure S1	Representative Slides for Each Stain

Supplementary Table S1. Antibodies and Staining Protocols.

ANTIBODY	HOST	COMPANY	PRODUCT CODE	DILUTION	DETECTION KIT
Collagen 1	rabbit	Abcam	ab34710	1/20000	IHC J 30 min; fast red
Collagen 3	mouse	Abcam	ab6310	1/50000	IHC J 30 min; fast red
CD68	mouse	Agilent	IR60961	Ready-to-use (RTU)	IHC J 30 min; fast red
CD117/c-kit	rabbit	Agilent	A4502	1/100	IHC J 30 min; fast red
ECP	mouse	Diagnostics Development	EG2	1/800	IHC J 30 min; fast red
MPO	rabbit	Agilent	IR51161	1/10	IHC J 30 min; fast red
CD8	mouse	Agilent	IR62361	Ready-to-use (RTU)	IHC J 30 min; fast red
CD4	mouse	Agilent	IR64961	Ready-to-use (RTU)	IHC J 30 min; fast red
CD20	mouse	Agilent	IR60461	Ready-to-use (RTU)	IHC J 30 min; fast red

Supplementary Figure S1. Representative Slides for Each Stain.

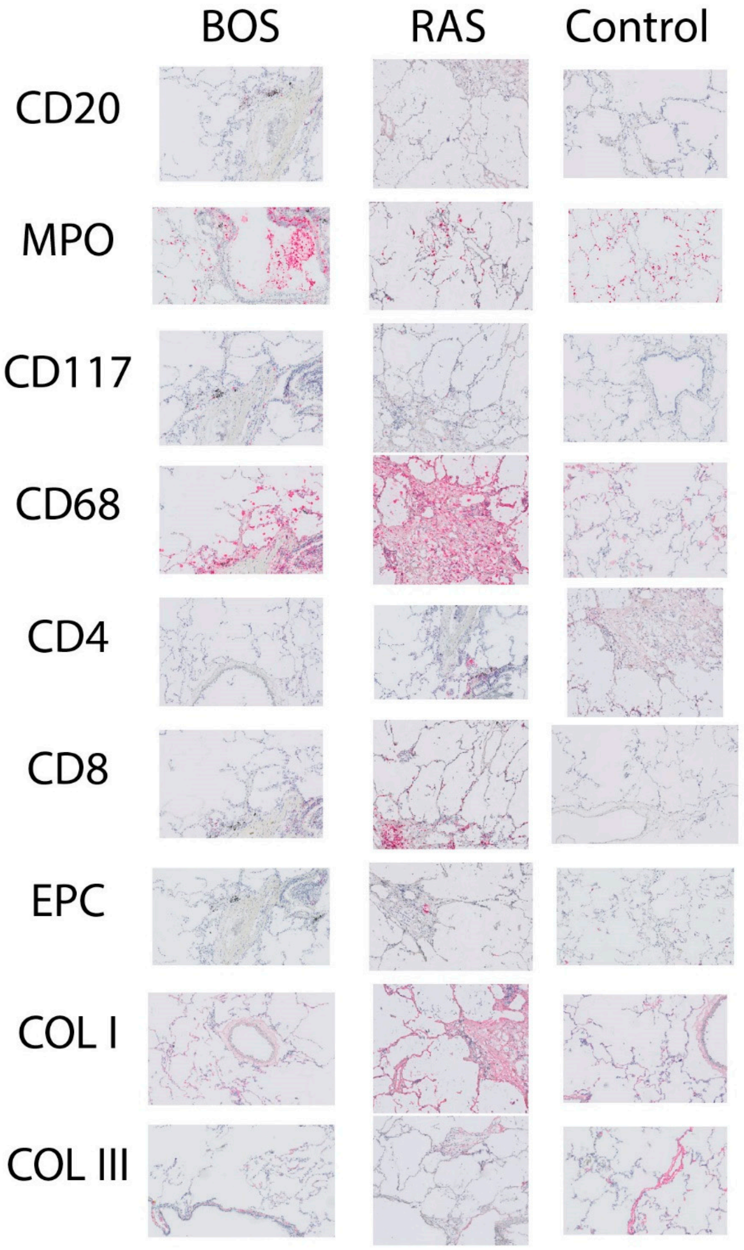

Legend: Representative slides were obtained for each stain from the BOS and RAS cases used in Figure 5, as well as for a control case. The control was an unused donor lung from a 37-year-old female.
